# Supplementary material for: Nonzero Berry phase in quantum oscillations from giant Rashba-type spin splitting in LaTiO3/SrTiO3 heterostructures
Source: Nat Commun. 2018 Apr 13;9:1458. doi: 10.1038/s41467-018-04014-0 (PMC5899139; doi:10.1038/s41467-018-04014-0)
Supplement: Supplementary file 1 — Supplementary Information [file 41467_2018_4014_MOESM1_ESM.pdf]

SUPPLEMENTARY NOTE 1. TEMPERATURE DEPENDENCE OF THE RESISTIVITY

Supplementary figure 1 shows the temperature dependence of the resistivity for LTO films of a wide range of thicknesses. As seen in this figure, the resistivity is thickness independent for films thicker than 2 nm, but increases dramatically when the film is only a few unit cells thick. Additionally, the ultra-thin films have a flatter temperature dependence than the thicker films. This indicates that there is likely a different source of conduction dominating the electrical transport in these ultra-thin films than the thick films.

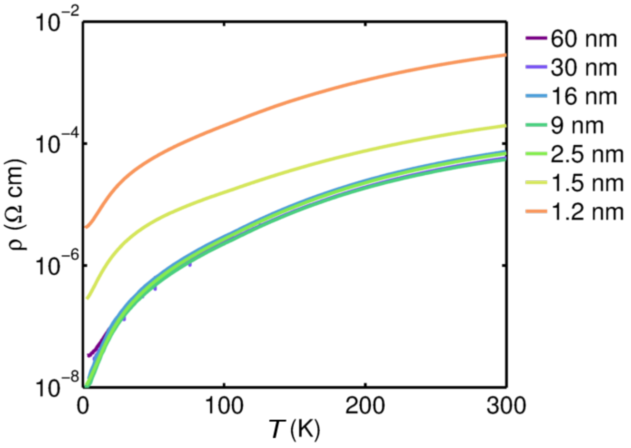

Supplementary Figure 1. **Resistivity temperature dependence** Temperature dependence of the resistivity for films of thicknesses ranging from 1.2 nm to 60 nm. The increase in resistivity for ultra-thin samples is clearly observed at all temperatures indicating the appearance of interfacial effects.

SUPPLEMENTARY NOTE 2. SHUBNIKOV-DE HAAS OSCILLATIONS WITH BACKGROUND SIGNAL REMOVED

We present the magnetic field dependence of the magnetoresistance data in Figure 1 of the main text. We expect the resistance oscillations to increase with increasing field if indeed these are Shubnikov de Haas oscillations. In order to demonstrate that the resistance oscillations are indeed increasing in amplitude as a function of magnetic field, we plot magnetoconductance as a function of magnetic field with the background removed. Supplementary figure 2 shows the background-removed low-frequency oscillations for the sample shown in Figure 1 of the text, and Supplementary figure 3 shows the high-frequency oscillations. The background was removed by fitting the magneto-conductivity to a cubic spline function, dividing by that function, and then subtracting 1.

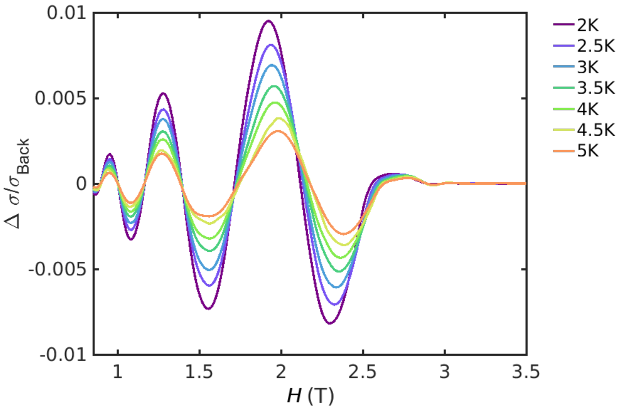

Supplementary Figure 2. **Isolated low frequency oscillations** Magnetoconductance data of Figure 1 with background removed to indicate the increasing amplitude of the oscillations with increasing magnetic field.

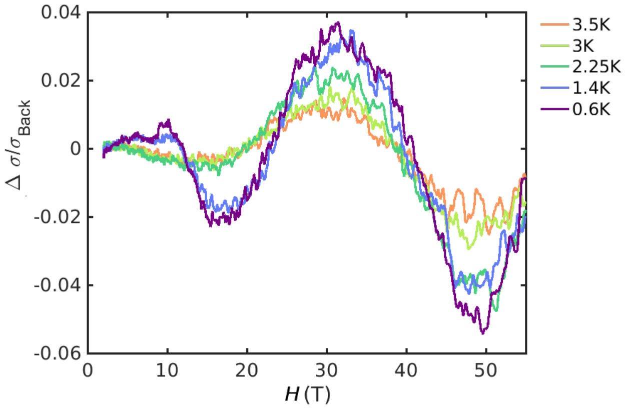

Supplementary Figure 3. **Isolated high frequency oscillations** Magnetoconductance data of Figure 2 with background removed. As is observed in the low frequency oscillations, the amplitude clearly also increases for the high frequency oscillations with increasing magnetic field.

SUPPLEMENTARY NOTE 3. FITTING THE OSCILLATIONS TO THE LIFSHITZ-KOSEVICH EQUATION

The magnetic field and temperature dependence of Shubnikov-de Haas oscillations is described by the Lifshitz-Kosevich equation,

$$\frac{\sigma_{xx}(H) - \sigma_{xx}(H=0)}{\sigma_{xx}(H=0)} \propto \sqrt{H}\frac{X(T)}{\sinh(X(T))}\exp(-X(T_D))\cos(2\pi(F/H + \phi_B - 1/2)), \quad (1)$$

where $\sigma_{xx}(H)$ is the longitudinal conductivity at a magnetic field, H, $X = (2\pi^2 m^* k_B T)/(\hbar e H)$, T_D is the Dingle temperature, m^* is the effective mass, and T is the temperature. This equation was used to fit the low frequency oscillations at 2K as shown in Supplementary figure 4 and the high frequency oscillations at 0.5K as shown in Supplementary figure 5.

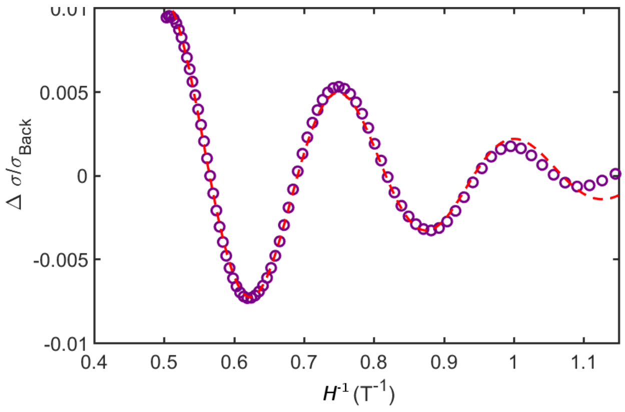

Supplementary Figure 4. **Low field Lifshitz-Kosevich fit** The background is removed from the low frequency fit to the Lifshitz-Kosevich equation. The fit is shown as a red dashed line. The data is well fit by the Lifshitz-Kosevich equation when a Berry phase of *pi* is taken into account.

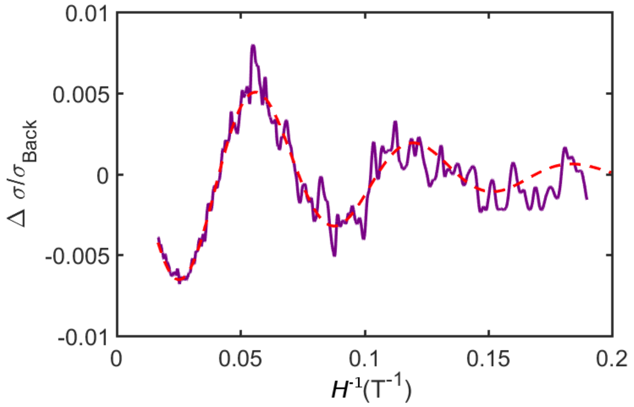

Supplementary Figure 5. **High field Lifshitz-Kosevich fit** The background is removed from the high frequency fit to the Lifshitz-Kosevich equation. The fit is shown as a red dashed line. The high frequency oscillations are well fit by the equation.

SUPPLEMENTARY NOTE 4. LAYER RESOLVED DENSITY OF STATES

Supplementary figures 6 and 7 show the contributions to the d_{xy}, d_{xz}, and d_{yz} orbitals from the different layers in the heterostructure. STO corresponds to the STO layer, IF is the interface layer, and 1LTO, 2LTO, and 3LTO are the three LTO monolayers. It is clear that the majority of the carriers and the Fermi energy which contribute to conduction come from the d_{xy} orbitals at the interface. However, very close to the Fermi surface is a d_{xz+yz} band which comes from the inner STO layer. We attribute the high-mobility carrier concentration observed in the Hall effect to the filling of this band.

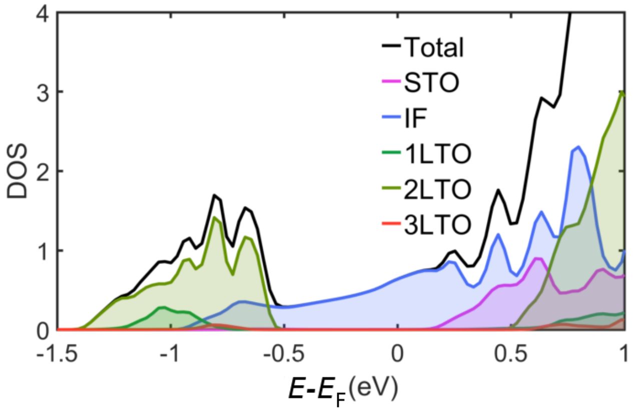

Supplementary Figure 6. d_{xy} **orbital density of states** Density of states for the d_{xy} orbitals for each of the layers in the heterostructure. There is clearly a large density of states at the Fermi energy due to the interfacial d_{xy} orbitals while all other layers form a gap at the Fermi energy. This density of states corresponds to band 2 of Figure 4 in the main text.

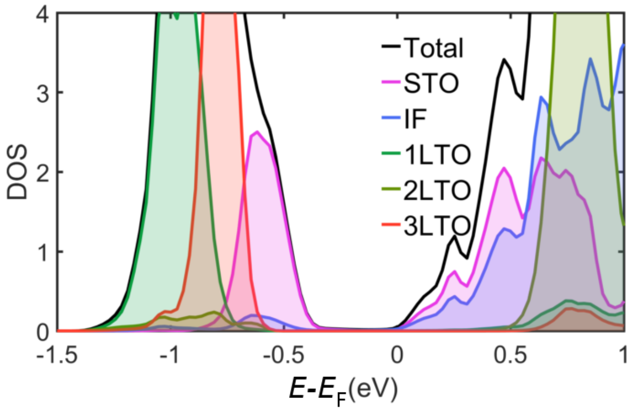

Supplementary Figure 7. d_{xz+yz} **orbital density of states** Density of states for the d_{xz+yz} orbitals for each of the layers in the heterostructure. Here the Fermi level is small but nonzero near the Fermi level, and it comes from the interfacial and STO derived d_{xz+yz} orbitals. This density corresponds to band 1 in Figure 4 of the main text.

SUPPLEMENTARY NOTE 5. DFT AND TIGHT BINDING MODEL COMPARISON

Supplementary figure 8 shows a close up of the tight binding model described in the main text along with the hybridized d_{xz+yz} band 1 from the DFT calculations along the $\Gamma - X$ and $\Gamma - M$ directions. As described in the main text, the tight binding hopping parameters were first adjusted to fit band 1 from the DFT calculations (light blue line in Supplementary figure 8) which produced the dashed black line in Supplementary figure 8. A linear Rashba term was then added to fit the area and cyclotron mass from the Shubnikov-de Haas oscillations to produce the red and blue curves.

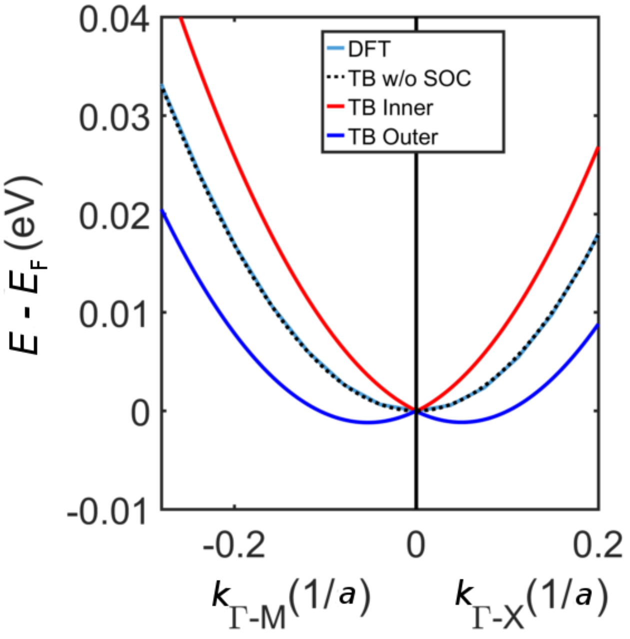

Supplementary Figure 8. **DFT and tight binding calculations** Comparison of the DFT calculations and the tight binding model along the $\Gamma - X$ and $\Gamma - M$ directions. The light blue light is the DFT calculated band, the dashed black line is the tight binding model without a Rashba term, and the red and blue lines are the tight binding model including a Rashba term.

SUPPLEMENTARY NOTE 6. FITTING THE HALL EFFECT TO A TWO BAND MODEL

The Hall data was fit to a two-carrier model using the zero field resistivity as an additional constraint. In this model, the magnetic field dependence of the Hall effect is given by

$$R_H = \frac{(\mu_1^2 n_1 + \mu_2^2 n_2) + (\mu_1 \mu_2 H)^2 (n_1 + n_2)}{e[(\mu_1 |n_1| + \mu_2 |n_2|)^2 + (\mu_1 \mu_2 H)^2 (n_1 + n_2)^2]}, \tag{2}$$

where μ_i is the mobility of the ith carrier, n_i is the carrier concentration of the ith carrier, e is the electron charge, and H is the magnetic field. Alone, this fit to the Hall effect cannot uniquely determine the mobilities and concentrations of the carriers. So to resolve this, we used the zero field constraint that the resistivity, ρ, is given $\rho = \frac{1}{e(n_1\mu_1 + n_2\mu_2)}$. This fit is shown in Supplementary figure 9 and gives estimates of two sheet carrier concentrations of $(1.9 \pm 0.2)10^{14}$ cm^{-2} and $(2.0 \pm 0.2)10^{12}$ cm^{-2} with mobilities of 46 ± 1 cm^2/Vs and $(4.0 \pm 0.1)10^3$ cm^2/Vs respectively at 10 K.

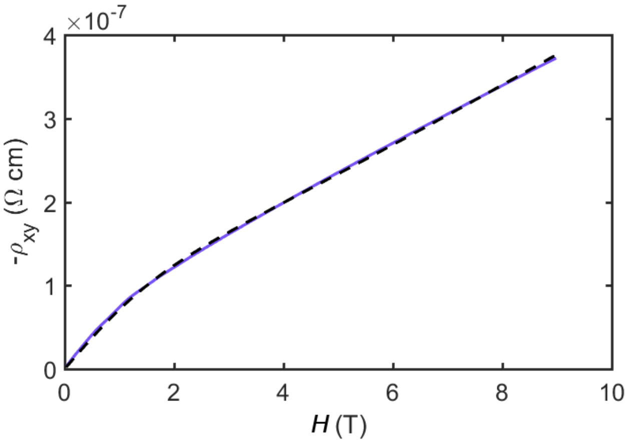

Supplementary Figure 9. **Nonlinear Hall effect fit** The Hall effect shown here is clearly nonlinear at 10 K. The fit to a two-band model is shown as a black dashed line. The parameters of this fit along with the zero field resistivity give the mobility and carrier concentrations given above and in the main text.

SUPPLEMENTARY NOTE 7. WEAK ANTI-LOCALIZATION FIT TO THE MAGNETOCONDUCTIVITY

We also found that the magnetconductivity data of our samples can be fit with a two dimensional weak anti-localization correction. A fit to the weak anti-localization model described in Hurand et al. enables us to extract a spin-orbit relaxation time from which we can deduce a Rashba coupling constant. This model is a modified version of the Maekawa-Fukuyama formulism with a quadratic orbital magnetoresistance term. Supplementary figure 10 shows the excellent fit of the data (show in solid orange) to the model (shown in dashed red). The Rashba constant from this fitting at 5 K is 2.0x10^{-11} eV-m in agreement with the value deduced from the two sets of quantum oscillations.

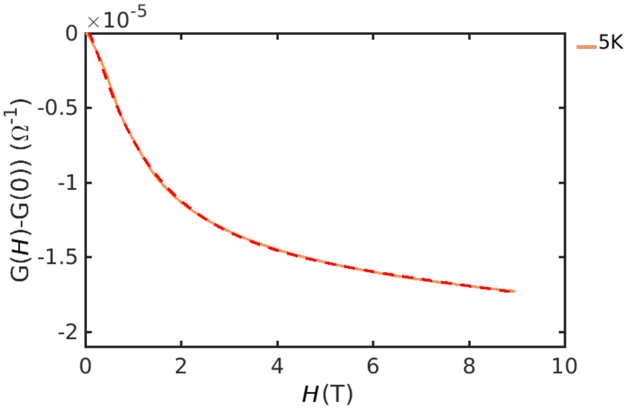

Supplementary Figure 10. **Weak anti-localization fitting** Magnetic field dependence of the conductivity is shown at 5K (solid orange line) along with a fit to a weak anti-localization model (dashed red line).

SUPPLEMENTARY NOTE 8. MAGNETIC FIELD DEPENDENCE OF THE IN-PLANE ANISOTROPIC MAGNETORESISTANCE

We have performed detailed anisotropic magnetoresistance (AMR) measurements as a function of both magnetic field and relative angle between the magnetic field and current directions. Supplementary figure 11 shows the magnetic field dependence for the in-plane anisotropic magnetoresistance as a function of the angle θ between the in-plane magnetic field and current shown in the schematic. The magnitude of the anisotropic magnetoresistance reaches a maximum around 2.5 T. Such a non-monotonic magnetic field dependence is consistent with numerical calculations for a Rashba system with a coupling constant and carrier concentration values similar to those obtained from our Shubnikov de Haas oscillations and weak anti-localization fitting.

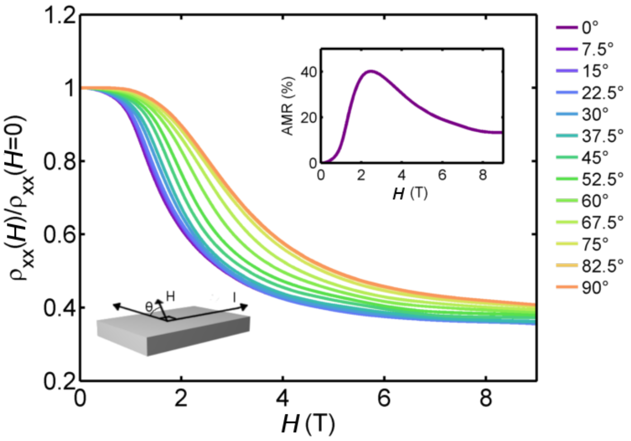

Supplementary Figure 11. **Field dependence of AMR** Magnetic field dependence of the magnetoresistance at specific in-plane angles between the magnetic field and current. The bottom-left inset shows a diagram of the setup and the definition of the in-plane angle θ. The top-right inset shows the strength of the AMR (see text for definition) as a function of magnetic field.
